# Tocopherol attenuates the oxidative stress of BMSCs by inhibiting ferroptosis through the PI3k/AKT/mTOR pathway

**DOI:** 10.3389/fbioe.2022.938520

**Published:** 2022-08-17

**Authors:** Dongmei Lan, Chao Yao, Xue Li, Haijiang Liu, Dan Wang, Yan Wang, Shengcai Qi

**Affiliations:** ^1^ Department of Prosthodontics, Shanghai Stomatological Hospital & School of Stomatology, Fudan University, Shanghai, China; ^2^ Shanghai Key Laboratory of Craniomaxillofacial Development and Diseases, Fudan University, Shanghai, China; ^3^ Medical College, Anhui University of Science and Technology, Huainan, China; ^4^ Department of Endodontics, Shanghai Stomatological Hospital & School of Stomatology, Fudan University, Shanghai, China; ^5^ Institute for Tissue Engineering and Regenerative Medicine, School of Biomedical Sciences, Ministry of Education Key Laboratory for Regenerative Medicine, Department of Orthopaedics and Traumatology, Faculty of Medicine, The Chinese University of Hong Kong, Hong Kong, Hong Kong SAR, China; ^6^ Center for Neuromusculoskeletal Restorative Medicine, Hong Kong Science Park, Hong Kong, Hong Kong SAR, China; ^7^ Department of Preventive Dentistry, Shanghai Stomatological Hospital & School of Stomatology, Fudan University, Shanghai, China

**Keywords:** BMSCs, tocopherol, PI3K/Akt/mTOR pathway, ferroptosis, osteogenic differentiation

## Abstract

Oxidative stress can induce bone tissue damage and the occurrence of multiple diseases. As a type of traditional medicine, tocopherol has been reported to have a strong antioxidant effect and contributes to osteogenic differentiation. The purpose of this study was to investigate the protective effect of tocopherol on the oxidative stress of rat bone marrow-derived mesenchymal stem cells (BMSCs) and the underlying mechanisms. By establishing an oxidative stress model *in vitro*, the cell counting kit-8 (CCK-8), reactive oxygen species (ROS) analysis, Western blot (WB), real-time PCR (RT-PCR), alkaline phosphatase (ALP) staining, and Alizarin Red staining (ARS) evaluated the effects of tocopherol on the cell viability, intracellular ROS levels, and osteogenic differentiation in BMSCs. In addition, ferroptosis-related markers were examined *via* Western blot, RT-PCR, and Mito-FerroGreen. Eventually, the PI3K/AKT/mTOR signaling pathway was explored. We found that tocopherol significantly maintained the cell viability, reduced intracellular ROS levels, upregulated the levels of anti-oxidative genes, promoted the levels of osteogenic-related proteins, and the mRNA of BMSCs stimulated by H_2_O_2_. More importantly, tocopherol inhibited ferroptosis and upregulated the phosphorylation levels of PI3K, AKT, and mTOR of BMSCs upon H_2_O_2_ stimulation. In summary, tocopherol protected BMSCs from oxidative stress damage *via* the inhibition of ferroptosis through the PI3K/AKT/mTOR pathway.

## 1 Introduction

Oxidative stress is considered as the phenomenon where the capacity of the antioxidant defenses is significantly lower than the ROS levels, resulting in potential damage in a biological system (Halliwell, 1996). As metabolic products by biological systems, ROS is mainly produced by mitochondria, which mainly include superoxide radicals (O2^•−^), hydrogen peroxide (H_2_O_2_), hydroxyl radicals (•OH), and singlet oxygen (^1^O_2_) (Navarro-Yepes et al., 2014). ROS is a double-edged sword for organisms. When maintained at an appropriate concentration, free radicals can participate in regulating intracellular signaling molecules and contribute to cell proliferation, differentiation, and self-renewability (Ludin et al., 2014). On the contrary, excess ROS plays an important role in pathogen resistance and cellular signaling, which is recognized as harmful reactive particles to damage cellular proteins, lipids, lipoproteins, and nucleic acids (Tan and Suda, 2018), which results in poor cell survival and oxidative damage. Many diseases are related to high ROS, such as inflammatory diseases and infection, neurodegeneration, cardiac diseases, diabetes, and osteoporosis (Forrester et al., 2018).

A growing number of studies have reported that the generation of ROS is associated with bone resorption. Patients with osteoporosis accumulate reactive oxygen species, which then leads to an increased level of oxidative stress (Agidigbi and Kim, 2019). High glucose conditions induce the production of NOX_2_-dependent cytosolic ROS in osteoclast activation *in vitro*. In streptozotocin-induced male diabetic rats, ROS upregulates the NLRP3 inflammasome response and enhances bone resorption by osteoclast activation, which may be an important cause for induced diabetic osteoporosis (An et al., 2019). Periodontitis is an inflammatory disease; the host immune response triggered by the Gram-negative anaerobic bacteria colonized on the subgingival plaque can result in an overproduction of ROS, further causing oxidative stress, apoptosis of periodontal ligament stem cells (PDLSCs), activation of osteoclasts, and resorption of alveolar bone (Sui et al., 2020). ROS generation can also activate P53/Bax-mediated apoptosis and mitochondrial dysfunction, leading to the reduction of osteogenic differentiation and an increase in bone loss (Li et al., 2015). Exposure to high levels of ROS can induce apoptosis of BMSCs *in vitro*. High levels of ROS lead to the poor survival rate of transplanted BMSCs in the ischemic tissue (Fang et al., 2019). Oxidative stress can inhibit the improvement of BMSC transplantation on the function of aging thymus and spleen (Wang et al., 2020). In addition, extensive ROS can interfere with the balance between the osteogenic and adipogenic differentiation of BMSCs, leading to severe bone diseases such as osteoporosis (Hu et al., 2021). When the osteoblasts are under oxidative stress, such as cells exposed to H_2_O_2_, ROS inhibits the secretory function of the osteoblasts (Atashi et al., 2015). The osteoclast activation dominates bone resorption, while the viability and function of BMSCs are key for bone regeneration. Therefore, it is necessary to find out a way to maintain the viability and function of BMSCs caused by oxidative stress in bone damage.

A variety of traditional Chinese medicines have obvious antioxidant effects, such as Hongjingtian (Zhao et al., 2021) and Salvia miltiorrhiza (Liang et al., 2021). The anti-oxidant tocopherol is a hydrolysis product of vitamin E that has the potential to scavenge free radicals. For example, tocopherol is able to prevent Alzheimer’s disease through its anti-oxidative effects (Dysken et al., 2014). Tocopherol protects porcine adipose-tissue–derived mesenchymal stem cells (pASCs) against H_2_O_2_-induced oxidative stress *in vitro* (Bhatti et al., 2018). Tocopherol has a protective effect on the cardiac function by inhibiting ischemia/reperfusion injury-induced oxidative responses (Wallert et al., 2019). More importantly, tocopherol plays a potentially positive effect on bone formation during the normal bone remodeling phase of secondary fracture healing, enhancing osseointegration of the implant to the bone *in vivo* (Savvidis et al., 2020). Tocopherol also improves osteoporosis by increasing the alkaline phosphatase activity in ovariectomized rats and prevents degenerative changes in trabecular bone structural parameters (Muhammad et al., 2012). In this study, we supposed that tocopherol might maintain the viability and function of BMSCs by decreasing the cellular level of ROS *in vitro*.

BMSCs will home and transfer to injured tissues and contribute to bone tissue repair. The overload and the toxic accumulation of ROS and lipid hydroperoxides in such high ROS conditions induce cell ferroptosis in bone metabolism (Yamada et al., 2020). Ferroptosis is an iron-dependent, non-apoptotic form of regulated cell death caused by lipid peroxidation, which is controlled by integrated oxidation and antioxidant systems. High glucose induces ferroptosis *via* increased ROS accumulation in type 2 diabetic osteoporosis (Ma et al., 2020). Therefore, inhibiting ferroptosis in BMSCs may potentially enhance the survival of BMSCs in oxidative stress. Tocopherol can efficiently suppress ferroptosis by neutralizing radical electrons (Fujii et al., 2022). Tocopherol plays cytoprotective effects in the prevention of ferroptosis against oxidative stress (Saito, 2021). Thus, we speculated that tocopherol protected BMSCs by inhibiting ferroptosis *via* anti-oxidative stress.

The aim of this study was to explore the roles of tocopherol in BMSCs under oxidative stress and the underlying molecular mechanisms. The cell viability of BMSCs under H_2_O_2_ stimulated was determined by CCK-8. The osteogenic differentiation capacity and ferroptosis of BMSCs were tested by ALP staining, Alizarin Red staining, RT-PCR, Western blot, and Mito-FerroGreen. Meanwhile, the PI3K/Akt/mTOR pathway was analyzed.

## 2 Materials and methods

### 2.1 Isolation and culture of BMSCs

BMSCs were harvested from 4-week-old male Sprague–Dawley (SD) rats. Briefly, the femurs were removed under aseptic conditions, and the bone marrow cavity was flushed out with an alpha minimum essential medium (α-MEM, Gibco, United States). After centrifuging at 1,000 rpm for 10 min, the cells were cultured in the α-MEM supplemented with 10% fetal bovine serum (FBS, Gibco, United States) and 1% penicillin–streptomycin (PS, Gibco, United States) at 37°C in a 5% CO_2_ incubator. Non-adherent cells were removed, and the medium was changed after 3 days. The cells obtained from passages 3 to 6 were used for further experiments.

### 2.2 Cell viability assay

The BMSCs were plated in 96-well plates (5 × 10^4^ cells/ml, 100 μl per well) and grown overnight. The cells were treated with 200 µM H_2_O_2_ and different concentrations of tocopherol (Sigma, United States) (from 1 to 200 µM) for 4 h. Then, each well was cultured with a normal medium for 24 h. The cell viability was stained with Cell Counting Kit-8 assays (CCK-8, Dojindo, Japan), and the OD value was determined at 450 nm by using a microplate reader (Bio-Rad, United States).

### 2.3 Analysis of the production of intracellular ROS

#### 2.3.1 Intracellular ROS measurement

The BMSCs were plated in 24-well plates (1 × 10^5^ cells/ml, 500 μl per well) for one night and incubated at 37°C in a serum-free medium containing 10 μM DCFH-DA for 20 min. Then, the cells were treated with 200 µM H_2_O_2_ and different concentrations of tocopherol (from 1 to 100 µM) for 4 h. Intracellular levels of ROS production were measured using a Reactive Oxygen Species Assay Kit (ROS Assay Kit, Beyotime, China) and imaged with a fluorescence microscope (Leica, Germany). The BMSCs were plated in 6-well plates (1 × 10^5^ cells/ml, 2 ml per well) for one night and incubated at 37°C in a serum-free medium containing 10 μM DCFH-DA for 20 min. Then, the cells were treated with 200 µM H_2_O_2_ and different concentrations of tocopherol (from 1 to 100 µM) for 4 h. Intracellular levels of ROS production were measured using a Reactive Oxygen Species Assay Kit and measured by flow cytometry (BD BioScience, United States).

#### 2.3.2 Analysis of the intracellular ROS-related mRNA level

The BMSCs were plated in 6-well plates (1 × 10^5^ cells/ml, 2 ml per well) and pre-treated with 200 µM H_2_O_2_ in a culture medium containing 10 µM tocopherol for 4 h. The expression levels of the anti-oxidative-related genes including *Nrf2, Cat, Sod-1,* and *Sod-2* were further confirmed. The RT-PCR process was performed as follows. The total mRNA was extracted by TRIzol (Invitrogen, United States) and then reverse-transcribed to cDNA using a PrimeScriptTM RT Master Mix (TaKaRa, Japan). DNA was probed by Hieff®qPCR SYBR Green Master Mix (Yeasen, Shanghai). With GAPDH serving as the endogenous control, a relative mRNA expression of the gene of interest was calculated using the comparative 2^–ΔΔCT^method. The primers were designed, as shown in Table 1.

**TABLE 1:** Nucleotide sequences of the primers used for RT -PCR.

Gene	Forward primer (5′-3′)	Reverse primer (5′-3′)
*Alp*	AAC​GTG​GCC​AAG​AAC​ATC​ATC​A	TGT​CCA​TCT​CCA​GCC​GTG​TC
*Opn*	CCATTTACGGAGACCCAC	TCTGAGCGGCAACTTTAT
*Ocn*	TGA​GGA​CCC​TCT​CTC​TGC​TC	GGG​CTC​CAA​GTC​CAT​TGT​T
*Runx2*	GCA​CCC​AGC​CCA​TAA​TAG​A	TTGGAGCAAGGAGAACCC
*Sod-1*	GCG​TCA​TTC​ACT​TCG​AGC​AG	ATA​GGG​AAT​GTT​TAT​TGG​GCA​ATC
*Sod-2*	GAG​CAA​GGT​CGC​TTA​CAG​A	CTC​CCA​GTT​GAT​TAC​ATT​CC
*Cat*	GTC​ACT​CAG​GTG​CGG​ACA​TTC	TCT​TAG​GCT​TCT​GGG​AGT​TGT
*Nrf2*	ATCTGAGTCCTTCACTG	GGG​ATA​CTG​TTC​ATC​AGA​AA
*Gpx4*	CGA​TAC​GCC​GAG​TGT​GGT​TT	CGG​CTG​CAA​ACT​CCT​TGA​TT
*Ptgs2*	TTC​CAA​ACC​AGC​AGG​CTC​AT	CAG​CGG​ATG​CCA​GTG​ATA​GA
*Ncoa4*	TCA​GAT​TGT​TAC​GGC​CTC​CC	GGT​CAC​TCA​GCT​CAC​GAT​GT
*Gapdh*	CAG​GGC​TGC​CTT​CTC​TTG​T	TCC​CGT​TGA​TGA​CCA​GCT​TC

### 2.4 Osteogenic differentiation of BMSCs

Osteogenic differentiation was induced by an osteogenic medium, which contains 10% FBS, 1% penicillin/streptomycin, 50 μg/ml ascorbic acid (Sigma, United States), 10 mM sodium β-glycerophosphate (Sigma, United States), and 10 nM dexamethasone (Sigma, United States). An osteogenic induction medium was used in the following experiment for 7 days (ALP staining and Western blot) and 14 days (Alizarin Red staining).

#### 2.4.1 Alkaline phosphatase and Alizarin Red staining

The BMSCs were plated in 24-well plates (1 × 10^5^ cells/ml, 500 µl per well) and pre-treated with 200 µM H_2_O_2_ and a culture medium containing different concentrations of tocopherol (from 1 to 100 µM) for 4 h. Then, each well was replaced with an osteogenic differentiation medium containing different concentrations of tocopherol for 7 and 14 days. The cells were fixed with 4% paraformaldehyde (Servicebio, Wuhan) and stained with ALP Staining (Beyotime, China) and Alizarin Red staining solution (Servicebio, Wuhan) for 15 and 30 min, followed by several washes with ddH_2_O. The osteogenic effects were observed and imaged by bright-field microscopy (Leica, Germany).

#### 2.4.2 Analysis of protein and mRNA levels

The BMSCs were plated in 6-well plates (1 × 10^5^ cells/ml, 2 ml per well) and pre-treated with 200 µM H_2_O_2_ and a culture medium containing 10 µM tocopherol for 4 h. The total proteins, including ALP, OPN, and RUNX2, were isolated for an osteogenic induction for 0 or 7 days with the RIPA lysis buffer (BioSharp, Shanghai) containing a protease inhibitor (BioSharp, Shanghai) and a phosphatase inhibitor (Beyotime, Shanghai) for Western blot. The protein concentration was measured using a BCA assay kit (Beyotime, Shanghai). The blots were probed with GAPDH (1:5,000; Abcam, United States), OPN (1:1,000; Abcam, United States), ALP (1:500; GenXspan, Shanghai), and RUNX2 (1:500; CST, United States) overnight at 4°C. The blots were washed and incubated for 2 h at room temperature with the HRP-conjugated secondary antibody (1:2000). The densitometry of the protein bands was quantified by Image J software. Osteogenic-related genes including *Alp*, *Opn*, *Ocn,* and *Runx2* were also analyzed by the aforementioned RT-PCR. The primers were designed, as shown in Table 1.

### 2.5 Accessibility of ferroptosis

#### 2.5.1 Analysis of protein and mRNA levels

The BMSCs were plated in 6-well plates (1 × 10^5^ cells/ml, 2 ml per well) and pre-treated with 200 µM H_2_O_2_ and a culture medium containing 10 µM tocopherol for 4 h. Ferroptosis-related proteins (GPX4, xCT, and ACSL4) and genes (*Gpx4*, *Nrf2*, *Ptgs2,* and *Ncoa4*) were tested by Western blot and RT-PCR, according to the aforementioned methods. ACSL4 (1:1,000) and xCT (1:1,000) were purchased from Boster (Boster, China). GPX4 (1:1,000) was purchased from Abmart (Abmart, Shanghai). The primers were designed as shown in Table 1.

#### 2.5.2 Mito-FerroGreen detection

The BMSCs were plated in a 24-well plate (1 × 10^5^ cells/ml, 500 µl per well). After the cells were grown overnight and after removing the medium, the cells were washed with a serum-free medium three times. Then, they were stained with a final concentration of 5 μM/L Mito-FerroGreen (Dojindo, Japan) for 30 min at 37°C and then washed with PBS three times again. Then, cells were cultured with 200 µM H_2_O_2_ and 10 µM tocopherol for 4 h. Images were immediately acquired using a confocal fluorescent microscope (Leica, Germany).

### 2.6 Pathway analysis

The BMSCs were plated in 6-well plates (1 × 10^5^ cells/ml, 2 ml per well) and pre-treated with 200 µM H_2_O_2_ and a culture medium containing 10 µM tocopherol for 4 h. Oxidative-related pathway proteins including P-PI3K (1:1,000), PI3K (1:1,000), P-AKT (1:1,000), AKT (1:1,000), P-mTOR (1:1,000), mTOR (1:1,000), P-AMPK (1:1,000), AMPK (1:1,000), P-P38 (1:1,000), P38 (1:1,000), P-ERK (1:1,000), and ERK (1:1,000) were tested. The BMSCs were plated in 6-well plates (1 × 10^5^ cells/ml, 2 ml per well) and pre-treated with 200 µM H_2_O_2_ and a culture medium containing 10 µM tocopherol and 5 µM ferrostatin-1 (MCE, United States) for 4 h The PI3K/AKT/mTOR pathway proteins including P-PI3K (1:1,000), PI3K (1:1,000), P-AKT (1:1,000), AKT (1:1,000), P-mTOR (1:1,000), and mTOR (1:1,000) were tested. All these proteins were purchased from Cell Signaling Technology (CST, United States).

### 2.7 Statistical analysis

A statistical analysis was performed by SPSS 20.0 (IBM, Somers, NY, United States). Each experiment was independently repeated at least three times. Differences between two groups were analyzed with an unpaired Student’s *t*-test, and more than three groups were analyzed with one-way analysis of variance, followed by the Bonferroni *post hoc* test. *p* values less than 0.05 were considered statistically significant. Results were expressed as means ± standard deviations (SD).

## 3 Results

### 3.1 Tocopherol protected H_2_O_2_-stimulated BMSCs by maintaining cell viability and reducing the ROS level

**FIGURE 1:**
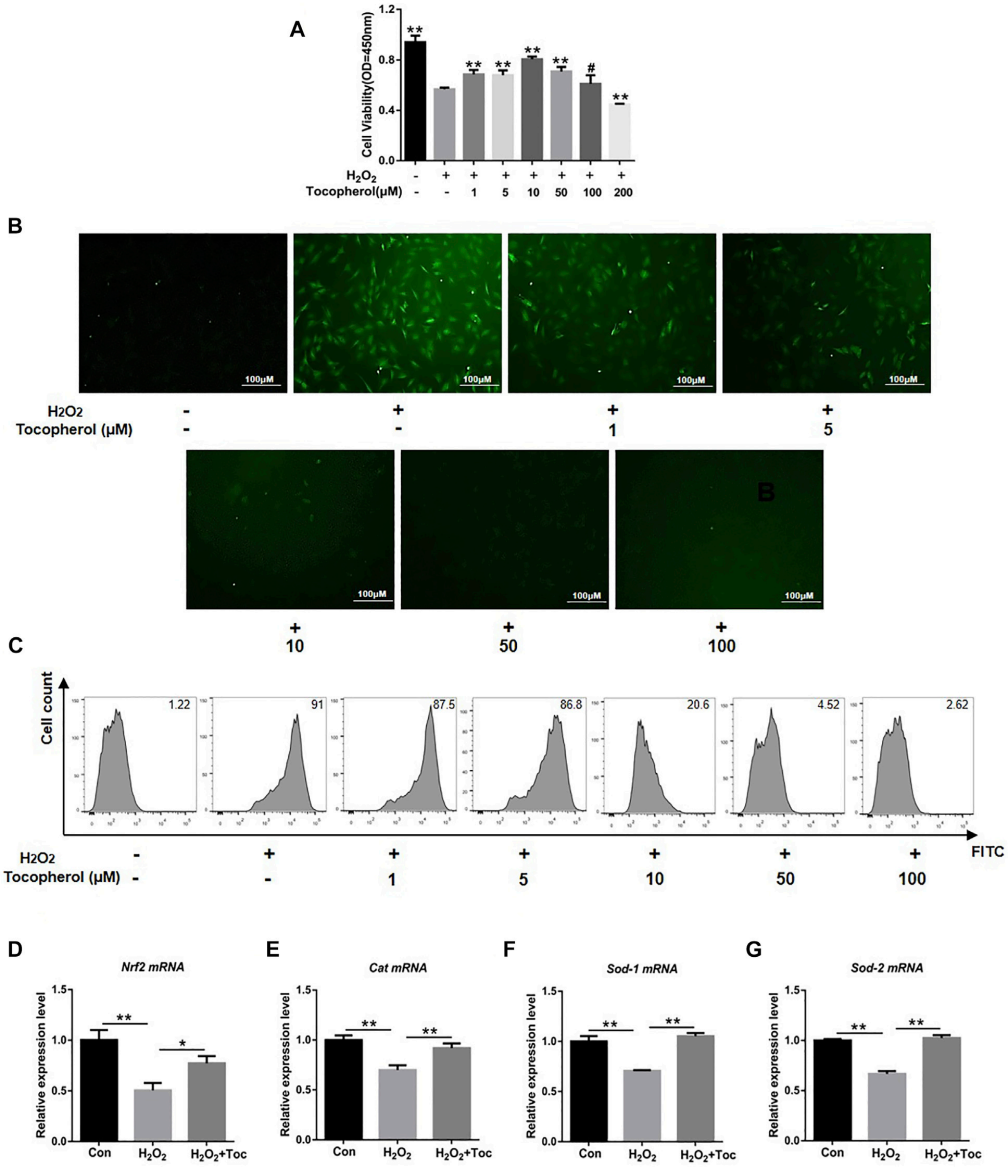
Assessment of the protection of different concentrations of tocopherol in H_2_O_2_-stimulated BMSCs. **(A)** Cell viability of H_2_O_2_-stimulated BMSCs after tocopherol (1–200 μM) treatment was detected by the CCK-8 assay. **(B)** Intracellular ROS measurement was measured by microscopy (scale bar = 100 μM). **(C)** Intracellular ROS measurement was measured by flow cytometry. **(D–G)** RT‐PCR analysis measured the ROS level of H_2_O_2_-stimulated BMSCs (*p < 0.05; **p < 0.01 vs. the H_2_O_2_ group).

To investigate the protective effect of tocopherol on H_2_O_2_-stimulated BMSCs, the cell viability was initially analyzed. As shown in Figure 1A, the cell viability of the 200 µM H_2_O_2_ group decreased significantly compared to the negative control group (without H_2_O_2_) (*p < 0.05*). Tocopherol at concentrations of 1, 5, 10, and 50 μM showed that the OD value was significantly higher than the positive control group (with H_2_O_2_) (*p < 0.05*), and the most appropriate concentration was 10 μM. ROS are important mediators of H_2_O_2_-induced cell death. In order to evaluate the effect of tocopherol on oxidative stress upon H_2_O_2_-stimulated BMSCs, the intracellular ROS level was tested. The data clearly suggested that the intracellular ROS level significantly increased upon exposure to H_2_O_2_, while tocopherol significantly attenuated the ROS production in a dose-dependent manner (from 1 to 100 μM), (Figures 1B,C; Supplementary Figure S1A). Meanwhile, the levels of anti-oxidative-related markers (*Nrf2*, *Cat*, *Sod-1*, and *Sod-2*) in 10 μM tocopherol-treated BMSC group were significantly upregulated, (Figures 1D–G) (*p < 0.05*). These results suggested that tocopherol maintained the cell viability and reduced the ROS level in H_2_O_2_-stimulated BMSCs *in vitro*.

### 3.2 Tocopherol maintained osteogenic differentiation capacity in H_2_O_2_-stimulated BMSCs

**FIGURE 2:**
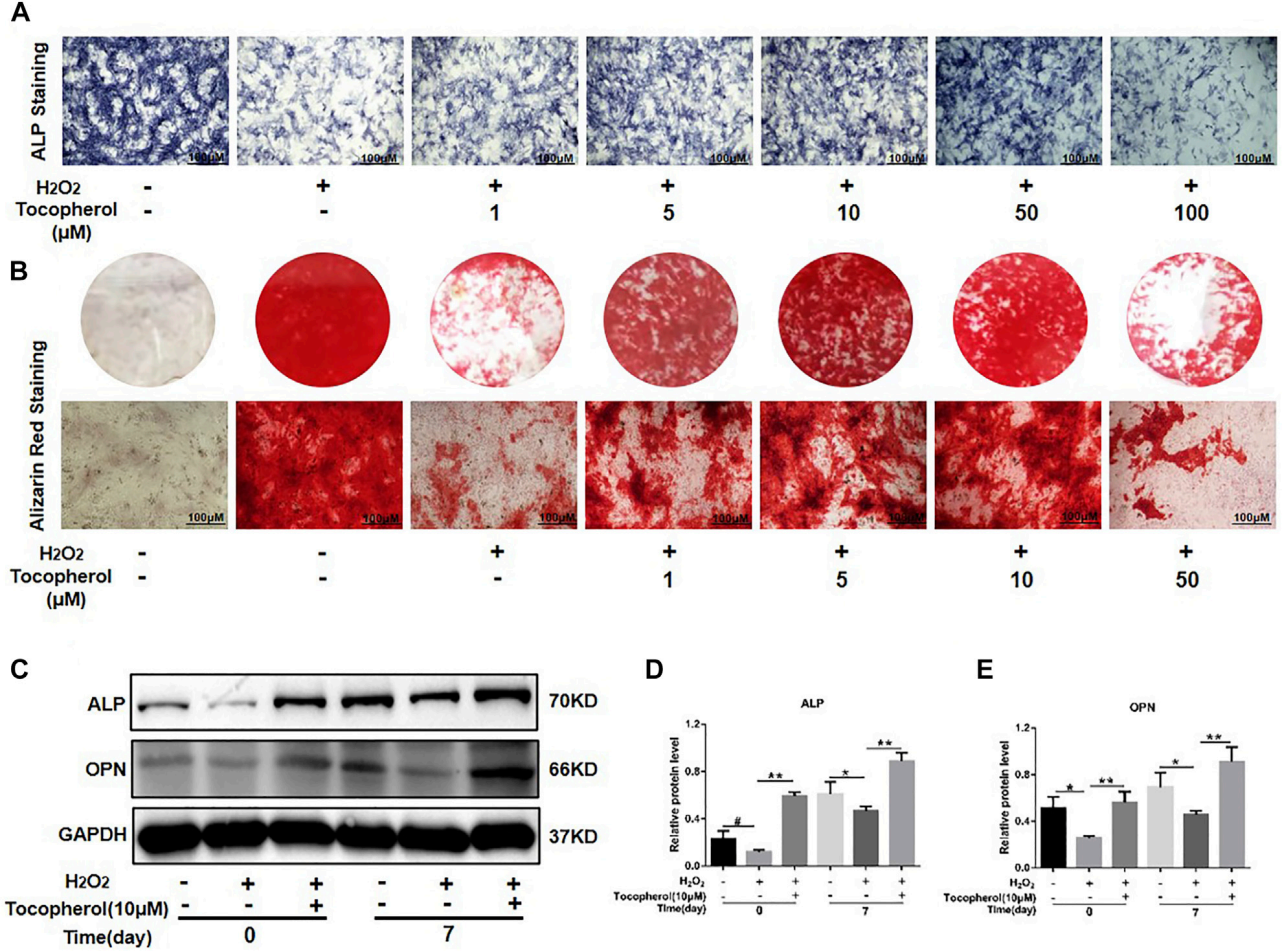
Effects of tocopherol on osteoblastic differentiation in H_2_O_2_-stimulated BMSCs. **(A)** BMSCs stained with ALP after 7 days of treatment (scale bar = 100 μM). **(B)** BMSCs stained with Alizarin Red after 14 days of treatment (scale bar = 100 μM). **(C)** Osteogenic-related protein expression of BMSCs was detected by Western blot after a treatment with H_2_O_2_ and tocopherol for 0 and 7 days. **(D–E)** Quantitative analysis of ALP and OPN.

The ALP activity and calcium deposition of BMSCs cultured with 200 μM H_2_O_2_ and different concentrations of tocopherol (from 1 to 100 μM) were examined. As shown in Figure 2 and Supplementary Figure S1B, ALP staining in the H_2_O_2_-treated groups was less intense than that in the negative control group, the ALP activity in the tocopherol group (from 1 to 50 μM) apparently increased, and was highest for the cells cultured with 10 μM tocopherol. For Alizarin Red staining, as shown in Figure 2B and Supplementary Figure S1B, the number of calcium deposition was notably decreased in the H_2_O_2_ group. Tocopherol (from 1 to 50 μM) contributed to the formation of calcium deposition of H_2_O_2_-stimulated BMSCs, especially at the concentration of 10 μM. In addition, H_2_O_2_ obviously decreased osteogenic differentiation, as indicated by the significantly reduced protein levels of ALP and OPN. However, the administration of tocopherol (10 μM) attenuated H_2_O_2_-suppressed BMSCs’ osteogenic differentiation, with significantly increased levels of osteogenic markers (ALP and OPN) (Figures 2C–E). These results suggested that tocopherol maintained the osteogenic differentiation of H_2_O_2_-stimulated BMSCs.

### 3.3 Tocopherol reversed H_2_O_2_-stimulated ferroptosis

**FIGURE 3:**
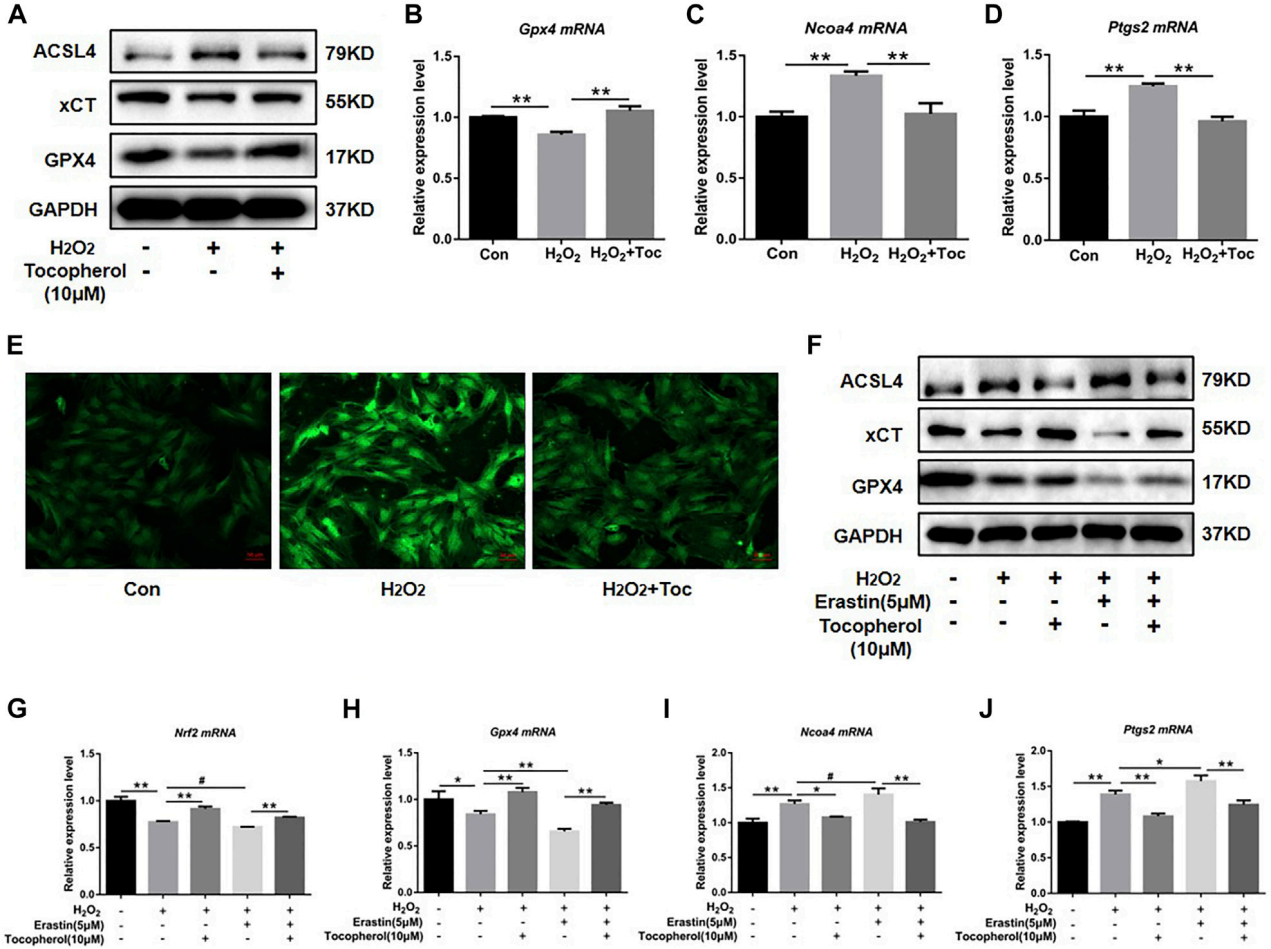
Effects of tocopherol on ferroptosis in H_2_O_2_-stimulated BMSCs. **(A)** GPX4, xCT, and ACSL4 protein levels in BMSCs were determined by Western blot. **(B–D)** *Gpx4*, *Ptgs2,* and *Ncoa4* mRNA levels in BMSCs were determined by RT-PCR. **(E)** Confocal fluorescent microscope was used to detect the generation of ferroptosis by the Mito-FerroGreen analysis. **(F–J)** Adding the ferroptosis activator erastin (5 μM) combined with H_2_O_2_ and tocopherol, ferroptosis-related proteins (ACSL4, xCT, and GPX4) and mRNA (*Gpx4*, *Nrf2*, *Ptgs2,* and *Ncoa4*) expression levels were analyzed. (*p < 0.05; **p < 0.01 vs. H_2_O_2_ group).

To explore the effect of tocopherol on ferroptosis, WB analysis and RT-PCR accompanied with the Mito-FerroGreen analysis were carried out. Compared with the negative group, the protein levels of GPX4 and xCT were downregulated, and ACSL4 was upregulated in the H_2_O_2_ group (Figure 3A; Supplementary Figure S1C). The mRNA levels of *Gpx4* and *Nrf2* were downregulated, and *Ncoa4* and *Ptgs2* were upregulated in the H_2_O_2_ group (Figures 3B–D) (*p < 0.05*). The confocal microscopy results showed that compared with the negative control group, the level of Fe^2+^ in the mitochondria in the positive group increased significantly (Figure 3E; Supplementary Figure S1D). These ferroptosis-related markers were reversed in the tocopherol-treated groups. The treatment of the cells with 5 μM erastin (ferroptosis agonist) considerably upregulated the ACSL4 protein expression and downregulated the GPX4 and xCT expressions (Figure 3F; Supplementary Figure S1E), upregulated the *Ptgs2* and *Ncoa4* mRNA expressions, and down-regulated the *Gpx4* and *Nrf2* expressions (Figures 3G–J) (*p < 0.05*). Moreover, these ferroptosis activation results could be partially reversed by tocopherol. All results suggested that tocopherol inhibited ferroptosis in H_2_O_2_-stimulated BMSCs.

### 3.4 Tocopherol promoted the osteogenic differentiation of H_2_O_2_-stimulated BMSCs by inhibiting ferroptosis

**FIGURE 4:**
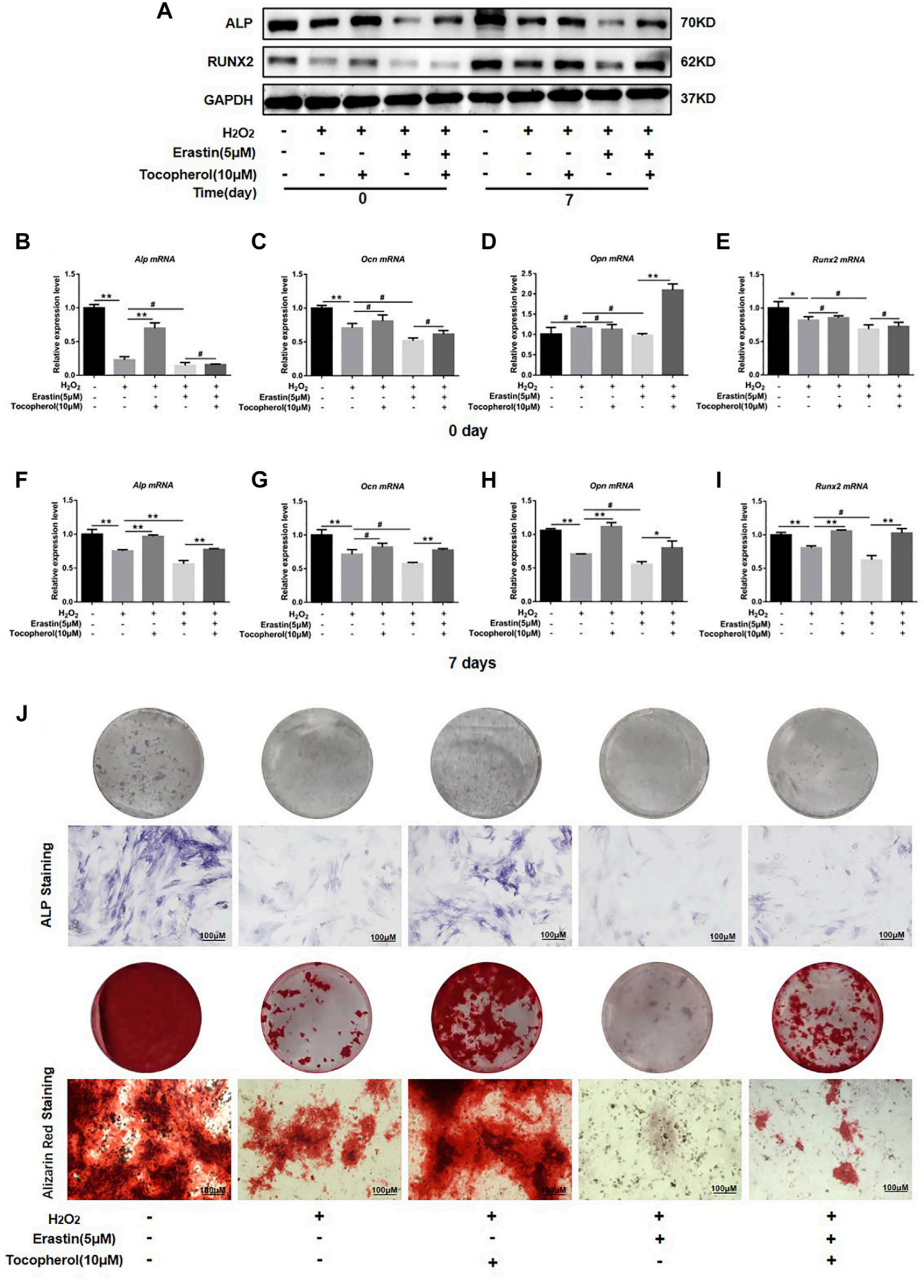
Effects of tocopherol on osteogenic differentiation in H_2_O_2_-stimulated ferroptosis. **(A)** ALP and RUNX2 protein levels were detected by Western blotting. **(B–I)** *Alp*, *Opn*, *Ocn*, and *Runx2* mRNA levels were detected by RT-PCR. **(J)** Osteogenic differentiation was evaluated by ALP staining and Alizarin Red staining. (*p < 0.05; **p < 0.01 vs. H_2_O_2_ group).

To investigate whether the effect of tocopherol on the osteogenic differentiation of H_2_O_2_-stimulated BMSCs *via* ferroptosis or not, the protein and mRNA levels of the osteogenic markers, ALP staining, and Alizarin Red staining were analyzed. The osteogenesis-associated protein (ALP and RUNX2) levels were downregulated in the erastin group, while the opposite trend was observed in both erastin and tocopherol groups at 0 and 7 days (Figure 4A; Supplementary Figure S1F). For further verification, we detected *Alp*, *Ocn*, *Opn,* and *Runx2* mRNA levels in BMSCs by RT-PCR. Compared with the erastin group, both erastin and 10 μM tocopherol significantly upregulated these osteoblastic-related mRNA expressions (Figures 4B–I) (*p < 0.05*). Additionally, ALP and Alizarin Red staining also showed that tocopherol promoted the mineralization of H_2_O_2_-stimulated BMSCs (Figure 4J; Supplementary Figure S1G). These results suggested that tocopherol inhibited ferroptosis to maintain the osteogenic differentiation of H_2_O_2_-stimulated BMSCs.

### 3.5 Tocopherol protected H_2_O_2_-stimulated BMSCs against ferroptosis *via* the PI3K/AKT/mTOR signaling pathway

**FIGURE 5:**
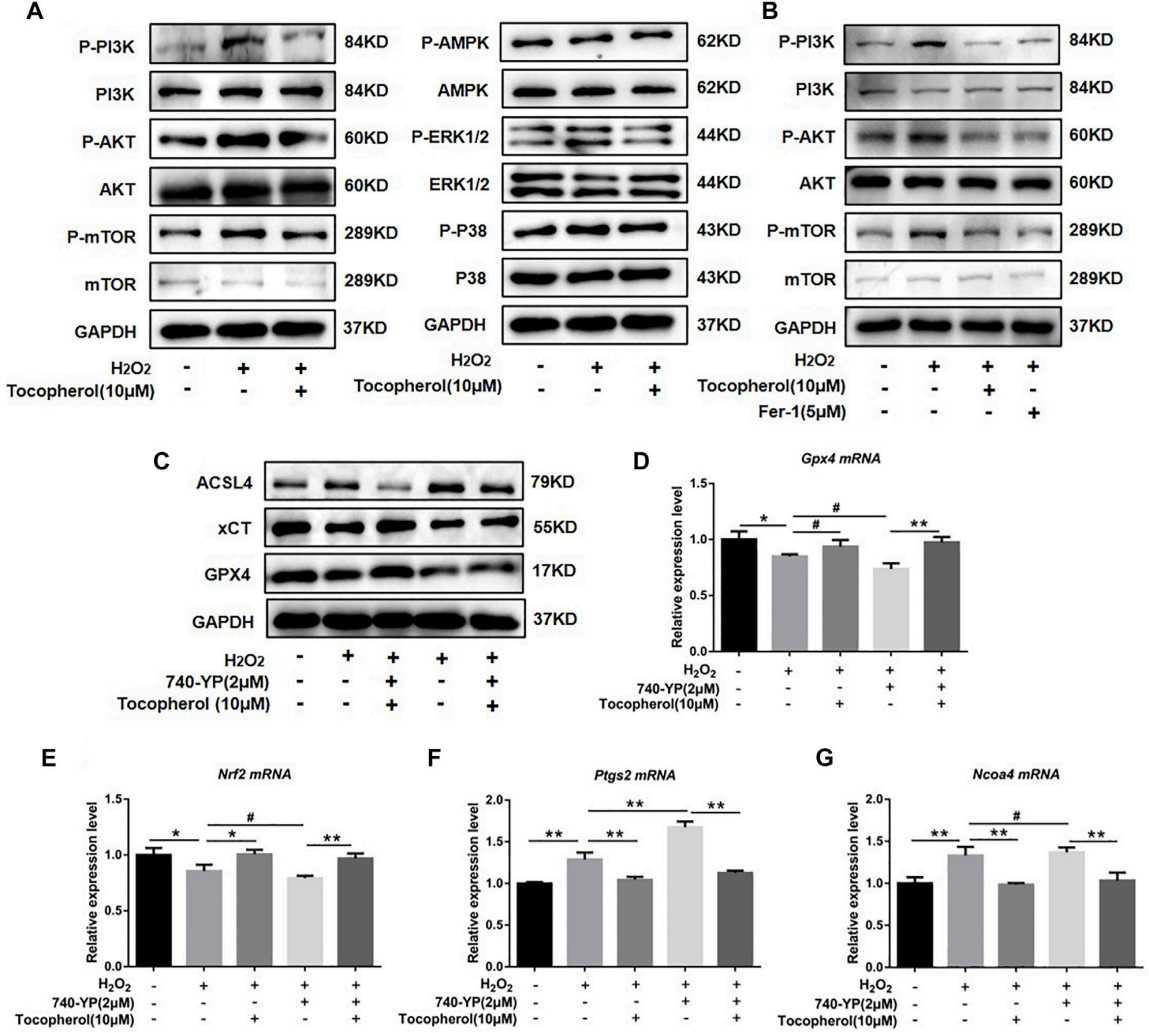
Exploration of the anti-oxidative stress mechanism of tocopherol. **(A)** Signaling pathways associated to oxidative stress were determined by Western blot. **(B)** Adding the ferroptosis inhibitor ferrostatin-1 (5 μM), PI3K/AKT/mTOR-related protein levels were determined by Western blot. **(C)** After treated with 2 μM 740-YP (a PI3K agonist), GPX4, xCT, and ACSL4 protein levels were determined by Western blot. **(D–G)** *Nrf2*, *Gpx4*, *Ptgs2*, and *Ncoa4* mRNA levels were determined by RT-PCR. (*p < 0.05; **p < 0.01 vs. H_2_O_2_ group)

To explore the mechanism by which tocopherol has an effect on antioxidants *via* ferroptosis, we analyzed several pathways associated to oxidative stress by Western blot, such as PI3K/AKT/mTOR, AMPK, and MAPK (P38 and ERK1/2). We found that H_2_O_2_ could notably upregulate the phosphorylation levels of PI3K, AKT, and mTOR. After tocopherol treatment, the phosphorylation levels of PI3K, AKT, and mTOR were downregulated, while no significant changes were found in other signaling pathways including AMPK and MAPK (P38 and ERK1/2) (Figure 5A; Supplementary Figure S2A). The synthetic antioxidant ferrostatin-1 also had a similar effect on the PI3K/AKT/mTOR pathway (Figure 5B; Supplementary Figure S2B). To further illustrate the role of the PI3K/AKT/mTOR pathway in H_2_O_2_-stimulated ferroptosis, the PI3K agonist 740-YP was used. The protein expressions of GPX4 and xCT were significantly downregulated, while ACSL4 was significantly upregulated after the 740-YP treatment (Figure 5C; Supplementary Figure S2C). Meanwhile, the mRNA expressions of *Gpx4* and *Nrf2* were downregulated, and *Ptgs2* and *Ncoa4* were upregulated after the 740-YP treatment. It was shown that the inhibition of ferroptosis by tocopherol was reversed after the 740-YP treatment (Figures 5D–G) (*p* < 0.05). The aforementioned results demonstrated that tocopherol potentially inhibited ferroptosis *via* inhibition of the PI3K/Akt/mTOR pathway.

### 3.6 Tocopherol promoted the osteoblastic differentiation of H_2_O_2_-stimulated BMSCs *via* the PI3K/AKT/mTOR pathway

**FIGURE 6:**
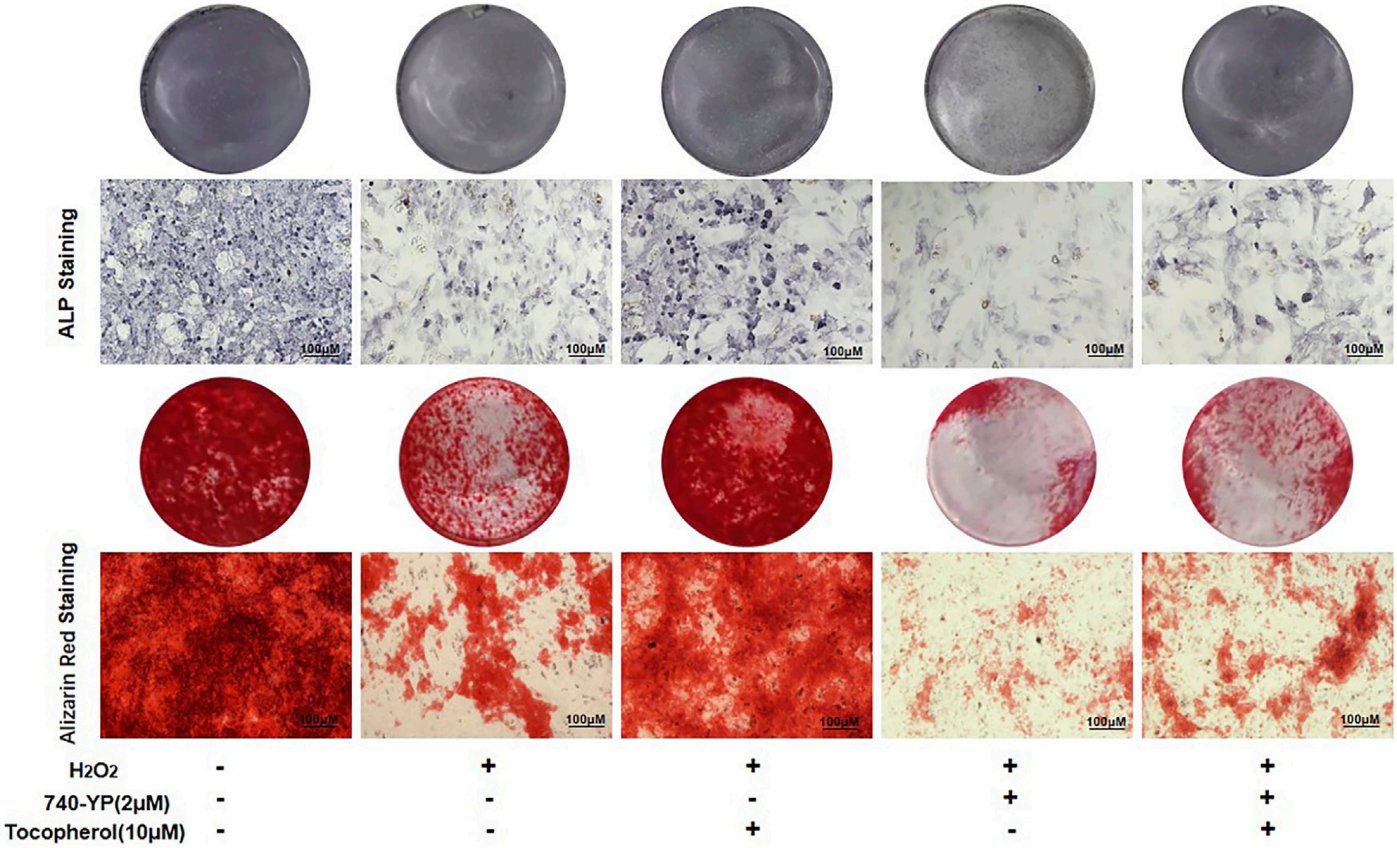
Regulation of osteogenic differentiation by tocopherol through the PI3K/AKT/mTOR signaling pathways. ALP and Alizarin Red staining of 10 µM tocopherol-treated BMSCs at day 7 and day 14, with or without 2 uM 740-YP for activating the PI3K/AKT/mTOR signaling pathway. (scale bar = 100 μM).

After 7 and 14 days of culture, the cells treated with 740-YP showed a decreased ALP activity and mineralized nodule formation compared with the H_2_O_2_ group, which obtained the reverse results in both the 740-YP and tocopherol groups (Figure 6, Supplementary Figure S2D). These results suggested that tocopherol might be positively correlated with the osteogenic ability of H_2_O_2_-stimulated BMSCs *via* the PI3K/AKT/mTOR pathway.

## 4 Discussion

Excessive intracellular levels of ROS are an important predisposing factor in multiple chronic diseases. In this study, tocopherol protected cell viability and osteoblastic differentiation of H_2_O_2_-stimulated BMSCs *via* anti-oxidative stress. Further mechanistic studies indicated the protective roles of tocopherol on H_2_O_2_-stimulated BMSCs *via* the inhibition of ferroptosis depending on the PI3K/Akt/mTOR signaling pathway, which provides a new strategy for the treatment of oxidative stress-related diseases.

From physiological levels, a low ROS level has a positive effect on proliferation and differentiation of cells (Le Belle et al., 2011). However, from pathological levels, a high ROS level causes lipid peroxidation and DNA fragmentation, ultimately leading to cellular death (Sun et al., 2015). It was reported that oxidative stress could shorten the telomere length of mesenchymal stem cells and inhibited their differentiation abilities. The increased level of ROS could promote phosphorylation of c-Jun N-terminal kinases to activate caspase-3, which in turn triggered the caspase cascade, leading to the apoptosis of BMSCs (Circu and Aw, 2010). In this study, the cell viability of H_2_O_2_-stimulated BMSCs decreased significantly, and the concentration of tocopherol from 1 uM to 50 uM significantly maintained cell viability of H_2_O_2_-stimulated BMSCs (Figure 1A). Meanwhile, tocopherol (10 uM) upregulated the expression of anti-oxidative-related genes (Figures 1D–G). These results indicated that tocopherol could significantly maintain cell viability by inhibiting intracellular oxidative stress. The effects of tocopherol on enhancing H_2_O_2_-stimulated osteogenic differentiation were confirmed by ALP staining and Alizarin Red staining (Figures 2A,B), and the expression of osteogenic-associated markers (Figure 2C). Above all, the results suggested that tocopherol could maintain the stemness of BMSCs by anti-oxidative stress.

Ferroptosis is an atypical form of programmed cell death associated with oxidative stress, iron accumulation, and lipid peroxidation. Ferroptosis is involved in the occurrence and development of many diseases, such as neurodegenerative disorders and bone metabolism diseases (Li et al., 2020). It was reported that the accumulation of ROS had been shown to activate the G protein axis and disrupted oxidative balance homeostasis to induce ferroptosis in BMSCs (Perillo et al., 2020), which affected the cell viability and reduced the survival rate after transplantation (Liu et al., 2022). Ferroptosis also could partly inhibit the activity of osteoblasts and affect its differentiation and mineralization process (Flood et al., 1989). High levels of butyrate had been found in the gingival crevicular fluids (GCFs) of patients with periodontitis, thus causing excessive accumulation of ROS and inhibiting the growth and proliferation of periodontal ligament fibroblasts (PDLFs), ultimately leading to ferroptosis (Zhao et al., 2020). Ferroptosis was also activated in H_2_O_2_-stimulated BMSCs in our study (Figure 3A). For further confirmation on the role of tocopherol on ferroptosis, erastin was used as a classical inducer of ferroptosis. The results showed that the activation effect of erastin on ferroptosis was partially reversed by tocopherol in H_2_O_2_-stimulated BMSCs (Figures 3F–J). Meanwhile, the inhibitory effect of erastin on osteoblast differentiation of H_2_O_2_-stimulated BMSCs was reversed by tocopherol (Figure 4). Overall, these results indicated that tocopherol inhibited ferroptosis of H_2_O_2_-stimulated BMSCs *in vitro*.

Oxidative stress has been reported to be involved in the regulation of multiple signaling pathways. For example, emodin could protect the hepatocyte cell line from paracetamol-induced oxidative damage by the activation of the AMPK pathway (Lee et al., 2020). Cyclosporin A had been shown to increase the viability of trophoblast cells and reduce the ROS-induced oxidative injury by the inhibition of the p38 pathway (He et al., 2020). Overexpression of Parkinson’s disease protein 7 prevented the oxidative stress-induced apoptosis of transplanted BMSCs by the activation of the ERK1/2 pathway (Zhang et al., 2021). Curcumin could attenuate oxidative stress and cardiomyocyte apoptosis to improve the cardiac function in streptozoticin-induced type 2 diabetic rats by the activation of the PI3K/AKT/mTOR pathway *in vivo* (Ren et al., 2020). Hyperactive mutation of PI3K/AKT/mTOR signaling can suppress cancer cells ferroptosis by upregulating the SREBP1-mediated lipogenesis. (Yi et al., 2020). In the present study, tocopherol significantly downregulated the phosphorylation levels of PI3K, AKT, and mTOR compared with BMSCs upon the H_2_O_2_-stimulation group, while there were no significant changes in other signaling pathways including AMPK and MAPK (P38 and ERK1/2) (Figure 5A). The effect of tocopherol on the PI3K/AKT/mTOR pathway was similar to ferrostatin-1 (Figure 5B). Subsequently, the results showed that treating BMSCs with the PI3K agonist (740-YP) effectively contributed to ferroptosis, which was inhibited after tocopherol treatment (Figures 5B–F). This indicated that ferroptosis was regulated by the PI3K/AKT/mTOR pathway in H_2_O_2_-stimulated BMSCs. In addition, the osteogenic differentiation of H_2_O_2_-stimulated BMSCs was specifically inhibited by 740-YP, which was reversed by tocopherol (Figure 6). All results confirmed that tocopherol had an effect on the anti-oxidative stress in BMSCs *via* the inhibition of ferroptosis through the PI3K/AKT/mTOR signaling pathway.

## 5 Conclusion

We collectively found that tocopherol improved the anti-oxidative stress processes in BMSCs by inhibiting ferroptosis *via* the PI3K/AKT/mTOR signaling pathway. It provided a theoretical basis for the future development of new drugs and guidance for the clinical application on oxidative stress-related diseases.

## Data Availability

The original contributions presented in the study can be directed to the corresponding author.
