# Erratum: Tocopherol attenuates the oxidative stress of BMSCs by inhibiting ferroptosis through the PI3k/AKT/mTOR pathway

**DOI:** 10.3389/fbioe.2023.1136971

**Published:** 2023-01-16

**Authors:** Frontiers Production Office

**Affiliations:** Frontiers Media SA, Lausanne, Switzerland

**Keywords:** BMSCs, tocopherol, PI3K/Akt/mTOR pathway, ferroptosis, osteogenic differentiation

Due to a production error, the author order in the correspondence section was incorrectly listed and author HL’s name was spelled incorrectly; “Shengcai Qi, Haijiang Li” was listed but instead, the author order and spelling should have been “Haijiang Liu, Shengcai Qi.”

The publisher apologizes for this mistake. The original version of this article has been updated.

