# Supplementary material for: Tocopherol attenuates the oxidative stress of BMSCs by inhibiting ferroptosis through the PI3k/AKT/mTOR pathway
Source: Front Bioeng Biotechnol. 2022 Aug 17;10:938520. doi: 10.3389/fbioe.2022.938520 (PMC9428255; doi:10.3389/fbioe.2022.938520)
Supplement: Supplementary file 1 [file Image1.pdf]

Supplementary Figure

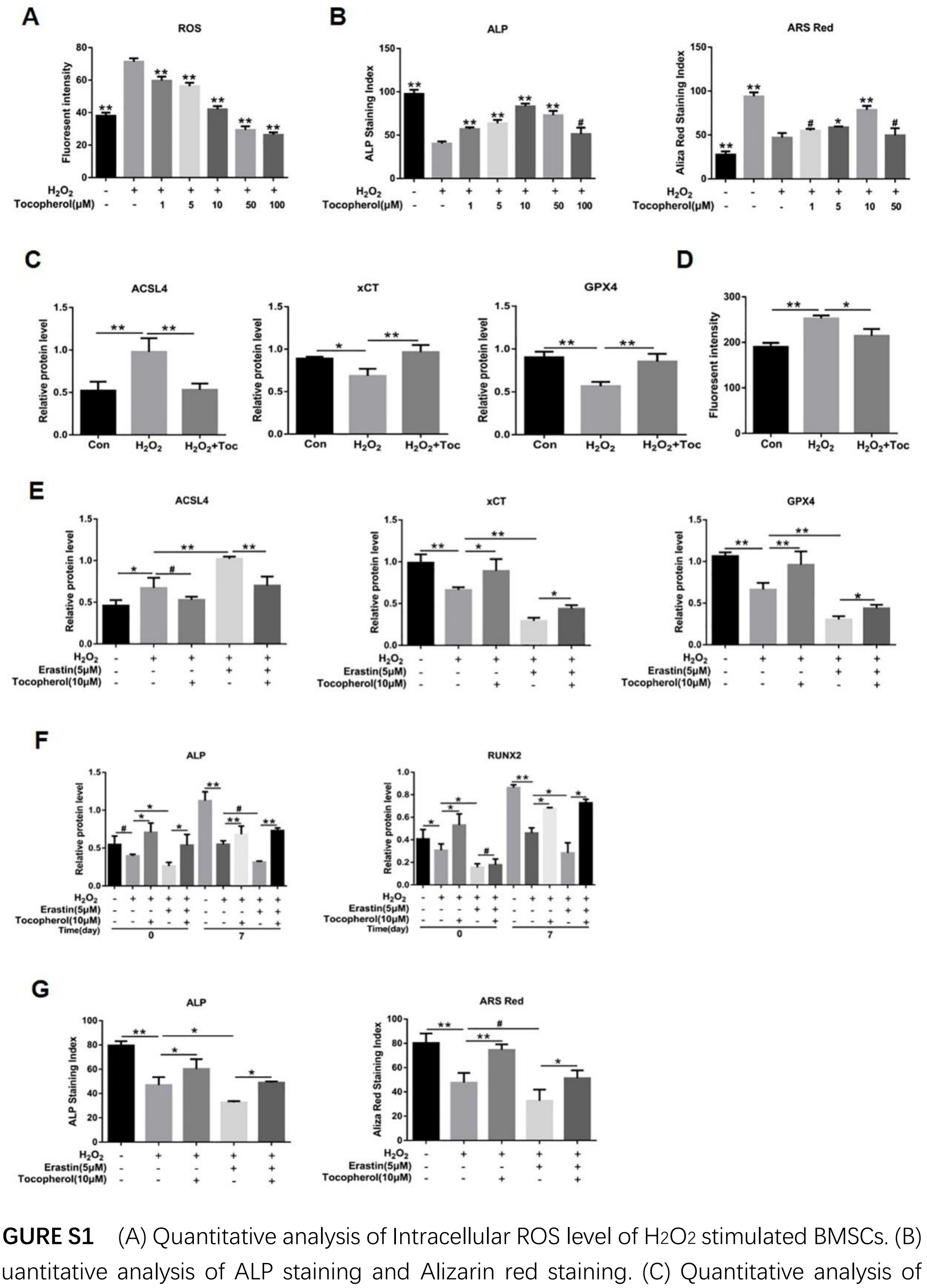

FIGURE S1 (A) Quantitative analysis of Intracellular ROS level of H_2O_2 stimulated BMSCs. (B) Quantitative analysis of ALP staining and Alizarin red staining. (C) Quantitative analysis of ferroptosis-related protein (ACSL4, xCT and GPX4) levels. (D) Quantitative analysis of the level of Fe^{2+} in the mitochondria. (E) Adding the ferroptosis activator Erastin (5 μM) combined with H_2O_2 and Tocopherol, the Quantitative analysis of ACSL4, xCT and GPX4 protein level. (F)

Quantitative analysis of osteogenesis-associated proteins ALP and RUNX2. (G) Adding the ferroptosis activator Erastin (5 μM) combined with H_2O_2 and Tocopherol, the Quantitative analysis of ALP staining and Alizarin red staining. (*p<0.05,**p<0.01 vs. H_2O_2 group)

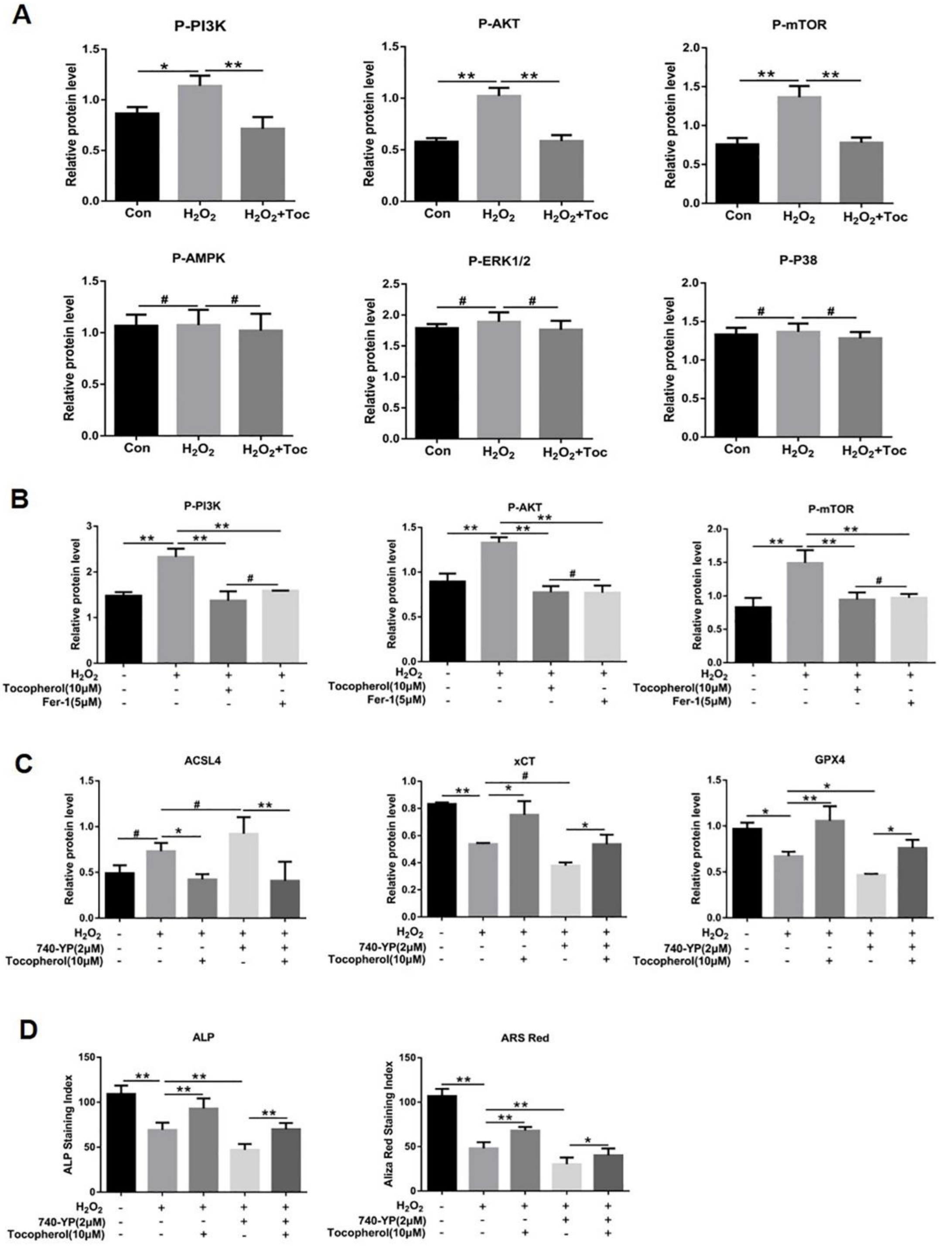

FIGURE S2 (A) Quantitative analysis of PI3K/AKT/mTOR, AMPK, MAPK (P38 and ERK1/2)-related protein levels. (B) Adding the ferroptosis inhibitor ferrostatin-1 (5 μM), the Quantitative analysis of PI3K/AKT/mTOR related protein levels. (C) Adding the the PI3K

agonist 740-YP (2 μM) combined with H_2O_2 and Tocopherol, the Quantitative analysis of ACSL4, xCT and GPX4 protein level. (D) Adding the the PI3K agonist 740-YP (2 μM) combined with H_2O_2 and Tocopherol, the Quantitative analysis of ALP staining and Alizarin red staining. (*$p<0.05$,**$p<0.01$ vs. H_2O_2 group)
